# Integrated Analysis of Paired Crude Oil and Produced Water Reveals Stress-Tolerant Hydrocarbon-Degrading Bacteria with Bioremediation Potential

**DOI:** 10.3390/microorganisms14091981

**Published:** 2026-09-08

**Authors:** Ning Mao, Tingli Yang, Ying Niu, Jingjing Zhang, Guike Yang, Xin Zhang, Xiaying Wang, Xiaobin Ou

**Affiliations:** Gansu Key Laboratory of Protection and Utilization for Biological Resources and Ecological Restoration in Longdong, School of Agriculture and Bioengineering, Longdong University, Qingyang 745000, China

**Keywords:** oilfield microbiome, cultivable bacteria, halotolerance, hydrocarbon removal, bacterial consortium

## Abstract

Oilfield microbiome studies have extensively characterized microbial community composition or isolated individual hydrocarbon degraders, but integrated exploration of cultivable functional bacteria from paired crude-oil and produced-water environments remains limited, particularly for strains combining hydrocarbon utilization with stress-tolerance and biosurfactant-related traits. In this study, paired crude-oil and produced-water samples from the Huoshaoshan Oilfield were investigated by combining 16S rRNA amplicon sequencing with culture-dependent isolation and functional screening. A total of 135 bacterial isolates representing 22 genera were recovered, of which 70 showed hydrocarbon-utilizing potential in primary screening. The collection also included isolates capable of growth at 10% NaCl and up to 55 °C. Comparison of sequencing and cultivation revealed marked differences between environmental abundance and recoverable functional resources. Representative isolates showed distinct removal profiles toward *n*-tetracosane (C_24_), naphthalene, phenanthrene, and fluoranthene under saline conditions, with *Halomonas nitritophilus* Ff1 and *Halomonas* sp. Fc15 showing particularly strong performance. Sequence-confirmed *alkB* and PAH-RHDα fragments provided additional molecular support for the observed hydrocarbon-removal phenotypes. A three-strain consortium composed of *Sphingomonas* sp. Fa24, *Halomonas nitritophilus* Ff1, and *Stutzerimonas stutzeri* Ff2 achieved 87.4% C_24_ removal, exceeding the corresponding monocultures. These findings link oilfield microbial community profiling with cultivable functional resource mining and provide candidate strains for saline petroleum bioremediation.

## 1. Introduction

Petroleum hydrocarbons remain major environmental contaminants associated with oil exploration, production, transportation, and refining [1]. Petroleum hydrocarbon contamination at concentrations above 20,000 mg kg^−1^ has been reported to markedly impair soil microbial activity and plant performance [2]. In heavily contaminated soils containing approximately 23,000–26,000 mg kg^−1^ petroleum hydrocarbons, substantial reductions in seedling emergence and soil-fauna survival have also been observed [3]. Their complex composition, hydrophobicity, persistence, and potential toxicity make the remediation of petroleum-contaminated environments challenging, particularly when conventional physicochemical treatments are costly or generate secondary waste [4]. Microbial biodegradation provides an economical and environmentally compatible alternative because indigenous microorganisms can transform or mineralize aliphatic and aromatic hydrocarbons through diverse metabolic pathways [5,6]. Petroleum-associated microorganisms are also of interest for reservoir-oriented applications, including microbial enhanced oil recovery (MEOR), in which microbial metabolites such as biosurfactants, organic acids, gases, and biopolymers may facilitate oil mobilization [7]. Biosurfactants (BS) are amphiphilic metabolites produced by microorganisms that reduce surface and interfacial tension at hydrocarbon–water interfaces, thereby facilitating emulsification, increasing hydrocarbon accessibility, and promoting oil mobilization in porous media [8]. In environmental contexts, these effects can improve the accessibility of hydrophobic petroleum hydrocarbons to microbial degraders and thereby facilitate biodegradation. In both environmental remediation and reservoir applications, microbial performance ultimately depends on the ability of functional populations to remain active under the physicochemical stresses of the target petroleum environment [9].

Salinity is a major constraint on microbial hydrocarbon degradation in oil reservoirs and produced water [10]. Elevated salt concentrations impose osmotic stress and can affect membrane integrity, enzyme activity, and microbial growth, thereby limiting the performance of conventional non-halotolerant degraders [11]. Consequently, halophilic and halotolerant hydrocarbon-degrading microorganisms have attracted considerable interest as microbial resources for saline petroleum environments. Members of *Halomonas*, *Marinobacter*, *Alcanivorax*, *Pseudomonas*, *Bacillus*, and related genera have been recovered from oilfields, saline soils, marine environments, and hypersaline habitats, with some strains retaining hydrocarbon-degrading activity at NaCl concentrations of 5–10%, and occasionally at even higher salinities [12,13,14]. Other physiological traits may further influence their application potential. Thermotolerance is advantageous in elevated-temperature petroleum systems, whereas biosurfactant production can increase the apparent bioavailability of poorly soluble hydrocarbons through emulsification and interfacial effects [15]. Accordingly, the recovery of hydrocarbon degraders that also possess stress-tolerance or biosurfactant-related traits is particularly desirable. Previous studies have reported individual strains and consortia combining some of these characteristics [8,16], but systematic characterization of cultivable oilfield microorganisms across multiple functional traits remains limited.

Oil reservoirs harbor specialized microbial communities shaped by temperature, formation-water salinity, pH, hydrocarbon composition, nutrient availability, and reservoir development history [17]. Produced water is therefore one of the most frequently investigated matrices in petroleum microbiology and commonly contains diverse hydrocarbon-degrading, fermentative, sulfate-reducing, and methanogenic populations [18,19]. In contrast, microorganisms directly associated with crude oil have received comparatively less attention, partly because of the technical challenges associated with low biomass and DNA recovery from the oil phase. Recent studies nevertheless indicate that crude oil can harbor distinct microbial assemblages influenced by petroleum composition, biodegradation, salinity, and reservoir operation [20,21]. Moreover, crude oil and produced water do not necessarily represent identical fractions of petroleum-associated microbial communities because microbial distribution may be influenced by phase partitioning, oil–water interfaces, injected water, and production processes [22,23]. Paired characterization of crude oil and corresponding produced water can therefore provide complementary information on reservoir-associated microbial resources, whereas such paired investigations remain less common than studies of produced water alone [21,24,25].

Studies combining molecular profiling with cultivation in petroleum-associated environments have shown that environmentally abundant taxa are not necessarily recovered proportionally in isolate collections, whereas selective cultivation can retrieve functionally relevant taxa that are poorly represented in sequencing datasets [21,26]. However, the relationship between environmental community composition and the recovery of stress-tolerant, hydrocarbon-utilizing bacteria remains insufficiently resolved, particularly when crude oil and corresponding produced water are considered together. A more integrated comparison of community profiles and cultivable functional resources may therefore provide a clearer assessment of petroleum-associated microbial potential [20,26,27].

Accordingly, we hypothesized that paired crude-oil and produced-water samples could provide complementary sources of cultivable functional bacteria and that environmental relative abundance does not necessarily predict the recovery of stress-tolerant hydrocarbon-utilizing isolates. To test this hypothesis, we aimed to (i) characterize the bacterial communities associated with the paired oilfield samples; (ii) recover and systematically phenotype cultivable bacteria for hydrocarbon utilization, halotolerance, thermotolerance, and oil-spreading activity; and (iii) quantitatively evaluate hydrocarbon removal by selected isolates and a consortium under saline conditions.

## 2. Materials and Methods

### 2.1. Chemicals and Culture Media

*n*-tetracosane (C_24_), naphthalene, phenanthrene, and fluoranthene were purchased from Macklin Biochemical Co., Ltd. (Shanghai, China). All other chemicals and medium components were purchased from Solarbio Science & Technology Co., Ltd. (Beijing, China), unless otherwise specified. Luria-Bertani (LB) medium contained 10.0 g/L tryptone, 5.0 g/L yeast extract, and 10.0 g/L NaCl. G3 mineral salts medium contained 5.0 g/L NaCl, 1.0 g/L K_2_HPO_4_, 1.0 g/L NH_4_H_2_PO_4_, 1.0 g/L (NH_4_)_2_SO_4_, 0.2 g/L MgSO_4_·7H_2_O, 3.0 g/L KNO_3_, pH 8.0–8.5, and 10 g/L crude oil, based on the formulation described by Sun et al. with modifications [28]. A high-salinity G2 medium contained 60.0 g/L NaCl, 1.0 g/L K_2_HPO_4_, 1.0 g/L NH_4_H_2_PO_4_, 1.0 g/L (NH_4_)_2_SO_4_, 0.2 g/L MgSO_4_·7H_2_O, 3.0 g/L KNO_3_, 1.0 g/L yeast extract, and 10 g/L crude oil. Basal mineral salts medium (MSM) was purchased from Sangon Biotech (Shanghai, China).

### 2.2. Sample Collection and Physicochemical Analysis

Three produced-water samples (W1–W3) and their corresponding crude-oil samples (O1–O3) were collected from different wells in the Huoshaoshan Oilfield, Xinjiang, China (89°1′16.18″ E, 44°56′34.31″ N). The crude-oil samples were obtained from wells H1469, H1380, and HTD2742, with average burial depths of 1480–1520, 1510–1550, and 1600–1650 m, respectively. The corresponding formation temperatures ranged from 61–63 °C to 65–67 °C, and the water cuts of the three wells were 86%, 76%, and 37%, respectively (Table S1). Samples were transported to the laboratory under refrigerated conditions and processed for subsequent analyses.

**Figure 1:**
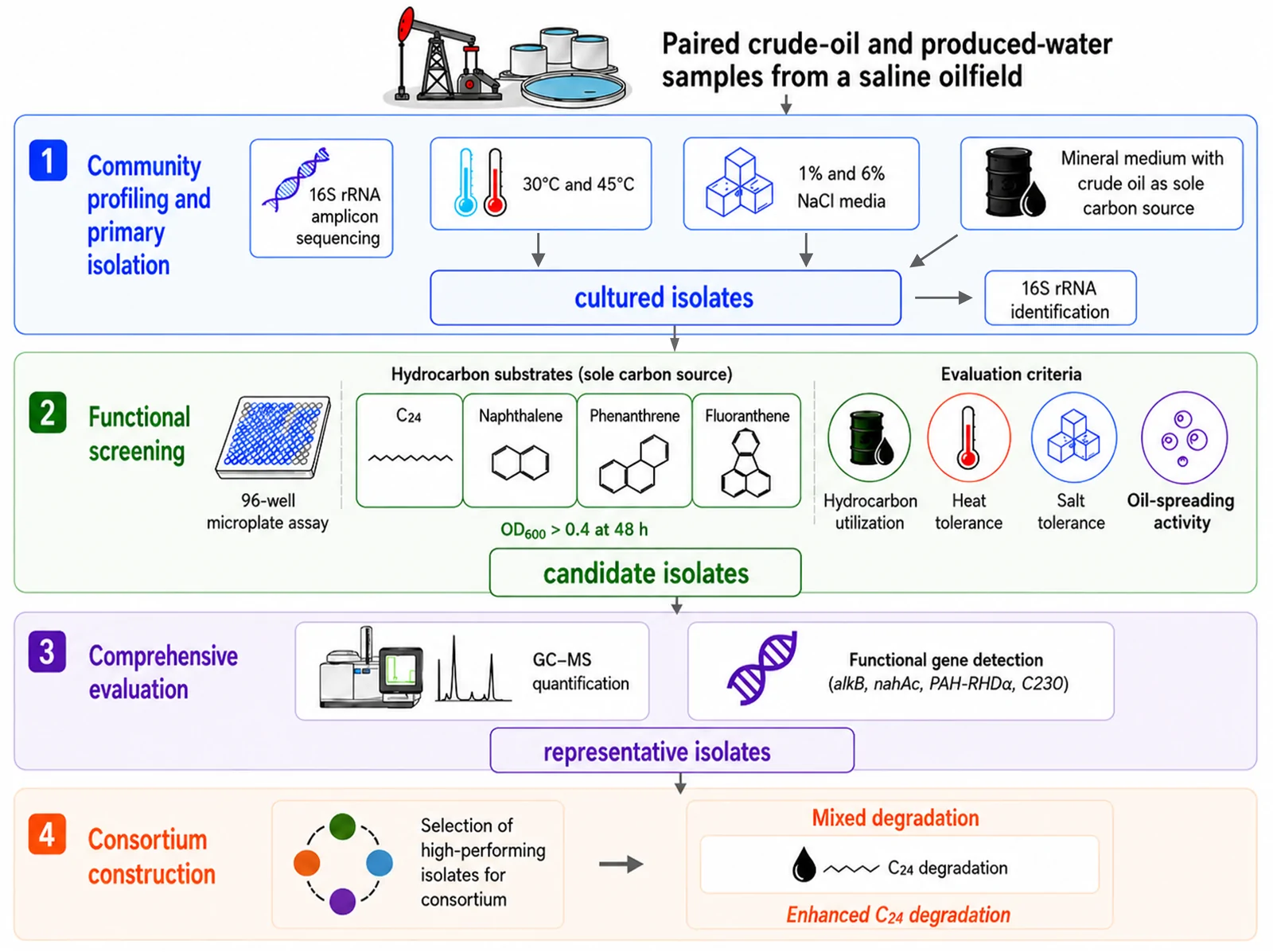
Overview of the experimental workflow.

The physicochemical characteristics of the produced-water samples were determined using standard analytical methods. Total dissolved solids (TDS) and total hardness (TH) were determined by the gravimetric method (DZ/T 0064.9-2021) [29] and EDTA titration method (DZ/T 0064.15-2021) [30], respectively. The permanganate index (CODMn) was measured by potassium permanganate titration according to GB/T 5750.7-2023 [31]. Total organic carbon (TOC) was determined using a TOC-L CPH total organic carbon analyzer (Shimadzu, Kyoto, Japan) in TC–IC mode. Total nitrogen (TN) and total phosphorus (TP) were measured by alkaline potassium persulfate digestion-UV spectrophotometry (HJ 636-2012) [32] and ammonium molybdate spectrophotometry (GB/T 11893-1989) [33], respectively. Total petroleum hydrocarbons (TPH) were quantified by a GC-MS-TQ8040 system (Shimadzu Corporation, Kyoto, Japan) with a capillary column (SH-Rxi-5Sil MS) following the method described previously by Mao et al. [34]. The complete physicochemical characteristics of the three produced-water samples are provided in Table S2. An overview of the experimental workflow is shown in Figure 1.

### 2.3. 16S rRNA Gene Amplicon Sequencing and Bioinformatic Analysis

Total microbial DNA was extracted from the six samples using the FastDNA SPIN Kit (MP Biomedicals, LLC, Irvine, CA, USA). The V3-V4 region of the bacterial 16S rRNA gene was amplified using primers 338F (5′-ACTCCTACGGGAGGCAGCAG-3′) and 806R (5′-GGACTACHVGGGTWTCTAAT-3′) [35]. PCR amplification was performed with an initial denaturation at 95 °C for 3 min, followed by 27 cycles of 95 °C for 30 s, 55 °C for 30 s, and 72 °C for 45 s, with a final extension at 72 °C for 10 min. PCR products were purified, quantified, pooled in equimolar amounts, and subjected to library preparation according to the standard Illumina amplicon-sequencing workflow by Majorbio Bio-Pharm Technology Co., Ltd. (Shanghai, China). The resulting libraries were paired-end sequenced on an Illumina NextSeq 2000 platform (Illumina, Inc., San Diego, CA, USA). The raw sequencing data were deposited in the NCBI Sequence Read Archive under accession number PRJNA1499344.

Raw reads were quality filtered and denoised, merged, and screened for chimeric sequences using DADA2 implemented in QIIME 2 (version 2020.2), and amplicon sequence variants (ASVs) were subsequently inferred. The optimized sequences, used for subsequent taxonomic assignment against the SILVA 138 database, varied in length from approximately 202 to 526 bp, with sample-specific mean lengths ranging from approximately 405 to 425 bp. Shannon diversity was calculated using the vegan package in R (version 4.3.1). Beta diversity among the six samples was visualized by PCoA based on Bray–Curtis dissimilarity, and differences between the produced-water and crude-oil matrices were evaluated by ANOSIM. Differentially represented taxa between the two matrix types were explored using LEfSe with an LDA score threshold of >2.0 and *p* < 0.05. At the genus level, taxa with a prevalence >0.8 within each matrix type were defined as core taxa.

### 2.4. Enrichment, Isolation, and Preliminary Screening of Cultivable Bacteria

For enrichment, 5 mL of each produced-water sample or 1 g of each crude-oil sample was separately inoculated into 100 mL of LB, G3, or G2 liquid medium. Cultures were incubated at 30 or 45 °C with shaking at 180 rpm. Every 7 d, aliquots of the enrichment cultures were transferred into fresh medium of the same composition. For subsequent transfers in G2 medium, yeast extract was omitted to increase selective pressure under high-salinity and hydrocarbon-containing conditions. After three successive transfers, the enrichment cultures were serially diluted and spread onto the corresponding solid media. Colonies differing in morphology, pigmentation, or size were selected and repeatedly streaked to obtain pure cultures.

Preliminary screening for hydrocarbon utilization was conducted in 96-well microplates. Each well contained 150 μL of MSM and was inoculated with 4 μL of an exponential-phase bacterial suspension that had been adjusted to an OD_600_ of 0.8 before inoculation. C_24_ was selected as a representative long-chain *n*-alkane, whereas naphthalene, phenanthrene, and fluoranthene were selected as representative two-, three-, and four-ring PAHs, respectively, to cover hydrocarbons with different structures and hydrophobicities. C_24_ was supplied at a final concentration of 0.1 g/L, whereas naphthalene, phenanthrene, and fluoranthene were each supplied at 0.01 g/L. C_24_ was dissolved in *n*-hexane, and the PAHs were dissolved in acetone before addition to the medium. Uninoculated substrate controls and solvent controls containing bacterial inoculum but no hydrocarbon were included to account for background absorbance and possible solvent effects on growth. Plates were incubated at 30 or 45 °C with shaking at 180 rpm, and growth was monitored by measuring optical density at 600 nm (OD_600_) using a BioTek microplate reader (BioTek Instruments, Winooski, VT, USA). Hydrocarbon utilization was scored according to OD_600_ after 48 h as follows: −, <0.1; +, 0.1–0.2; ++, 0.2–0.3; +++, 0.3–0.4; and ++++, >0.4. Isolates reaching an OD_600_ of at least 0.3 with one or more hydrocarbons were classified as hydrocarbon-utilizing isolates. Isolates reaching an OD_600_ above 0.4 after 48 h with at least one hydrocarbon were subjected to further multistress phenotyping.

### 2.5. Halotolerance and Thermotolerance Assays

Halotolerance was evaluated in LB medium containing 1%, 2%, 4%, 6%, 8%, or 10% NaCl (*w*/*v*). Each well of a 96-well plate contained 150 μL of medium and 4 μL of an exponential-phase bacterial suspension adjusted to an OD_600_ of 0.8. Uninoculated medium of the same composition served as the blank control. All assays were performed in triplicate. Plates were incubated at 30 or 45 °C with shaking at 180 rpm for 48 h, and OD_600_ was measured periodically. Growth at a given NaCl concentration was considered positive when OD_600_ > 0.3 after 48 h.

The isolates initially recovered at 45 °C were further evaluated for thermotolerance at 45, 50, 55, and 60 °C. A 4 μL aliquot of bacterial suspension adjusted to an OD_600_ of 0.8 was added into 150 μL of LB medium. Cultures were incubated at 45, 50, 55, or 60 °C with shaking at 180 rpm for 48 h. OD_600_ was measured at 6 h intervals, and uninoculated LB medium served as the blank control. All assays were conducted in triplicate.

### 2.6. Oil-Spreading Assay

The oil-spreading assay was performed using a modified method based on Morikawa et al. [36]. Oil-spreading activity was evaluated using cell-free culture supernatants. A 1 mL aliquot of bacterial culture was centrifuged at 8000 rpm for 5 min, using an Eppendorf Centrifuge 5420 (Eppendorf SE, Hamburg, Germany), and the supernatant was collected. A 20 mL volume of distilled water was added to a 90 mm Petri dish, followed by 1 mL of Sudan III-stained liquid paraffin prepared from 0.1 g Sudan III in 20 mL liquid paraffin. After formation of a uniform oil layer, 10 μL of culture supernatant was gently applied to the center of the plate. The diameter of the resulting clear zone was measured after several seconds. Oil-spreading activity was classified according to clear-zone diameter as −, <0.5 cm; +, 0.5–1.0 cm; ++, 1.0–2.0 cm; +++, 2.0–3.0 cm; and ++++, >3.0 cm.

### 2.7. Molecular Identification of Cultured Isolates

Genomic DNA from all purified isolates was subjected to PCR amplification of the nearly full-length 16S rRNA gene using primers 27F (5′-AGAGTTTGATCMTGGCTCAG-3′) and 1492R (5′-TACGGYTACCTTGTTACGACTT-3′) [37]. PCR products were sequenced by Sanger sequencing, and the resulting sequences were compared with reference sequences in the NCBI database using the online BLASTntool (NCBI, Bethesda, MD, USA). The best-fit nucleotide substitution model was selected in MEGA 12 according to the Bayesian Information Criterion (BIC). The General Time Reversible model with a gamma distribution (GTR+G) was identified as the best-fitting model and was used to construct a Maximum Likelihood phylogenetic tree. Branch support was evaluated with 1000 bootstrap replicates. The 16S rRNA gene sequences of the 135 cultured isolates have been deposited in GenBank under accession numbers PZ692254-PZ692388 (Table S4).

### 2.8. Quantitative Hydrocarbon Removal and GC-MS Analysis

Bacterial cultures grown to the exponential phase were centrifuged, and the harvested cells were washed two to three times with phosphate-buffered saline (PBS). The washed cells were resuspended and inoculated at 1% (*v*/*v*) into 100 mL MSM supplemented with 6% NaCl. Hydrocarbon concentrations and solvent carriers were the same as those used in the preliminary screening described in Section 2.4. Sterile medium containing the corresponding hydrocarbon but no bacterial inoculum served as the abiotic control. Cultures were incubated at 30 or 45 °C with shaking at 180 rpm for 7 d. All hydrocarbon-removal assays were performed in triplicate.

After incubation, C_24_ was extracted with an equal volume of *n*-hexane, whereas PAHs were extracted with an equal volume of ethyl acetate. The organic phases were dried over anhydrous sodium sulfate and concentrated by rotary evaporation at 45 °C. C_24_ extracts were brought to a final volume of 10 mL with *n*-hexane, whereas PAH extracts were adjusted to 10 mL with acetone.

Hydrocarbons were quantified using an Agilent 7890B gas chromatograph coupled to a 5977A mass selective detector (Agilent Technologies, Santa Clara, CA, USA) and equipped with a DB-5MS capillary column (30 m × 0.25 mm × 0.25 μm). The oven temperature was initially held at 50 °C for 2 min, increased to 300 °C at 10 °C/min, and held at 300 °C for 5 min. High-purity helium was used as the carrier gas at a flow rate of 1.0 mL/min. Mass spectra were acquired under electron-impact ionization at 70 eV over an m/z range of 50–550. Hydrocarbon concentrations were quantified using external calibration curves (Figure S2), and removal efficiency was calculated relative to the abiotic control.

### 2.9. Detection and Sequence Verification of Hydrocarbon-Degradation-Related Genes

Five published primer sets targeting hydrocarbon-degradation-related genes were used, including alkB-1 [38], ALK3 [39], PAH-RHDα [40], nahAc [41], and C23O [42]. Primer sequences are listed in Table 1. PCR products of the expected size were purified and subjected to Sanger sequencing. The resulting sequences were analyzed using the online BLASTxtool (NCBI, Bethesda, MD, USA) to verify target identity. The sequence-confirmed *alkB* fragments from *Sutcliffiella cohnii* Fa8 and *Halomonas nitritophilus* Ff1 have been deposited in GenBank under accession numbers PZ815455 and PZ815456, respectively. The PAH-RHDα sequence from *Sphingomonas* sp. Fa24 has been deposited under accession number PZ815457.

### 2.10. Three-Strain Consortium C_24_ Removal Assay

The three isolates showing the highest individual C_24_ removal efficiencies were selected for consortium testing. Pairwise antagonistic interactions among the three consortium members were evaluated using a cross-streak assay with minor modifications [43]. Each strain was first streaked on LB agar and pre-incubated at 30 °C for 10–12 h. The other two strains were then streaked perpendicular to the pre-grown strain and extended toward its growth margin. Plates were further incubated at 30 °C, and antagonism was assessed based on visible inhibition of growth adjacent to the opposing streak. Each strain pair was tested in both directions. Each strain was cultivated separately to the exponential phase, harvested by centrifugation, washed two to three times with PBS, and resuspended to an OD_600_ of 0.8. Equal volumes of the three suspensions were mixed at a 1:1:1 ratio. The consortium was inoculated at a total inoculum of 1% (*v*/*v*), identical to that used for each monoculture treatment. An uninoculated abiotic control was included. Hydrocarbon addition and incubation conditions were the same as those described in Section 2.8.

### 2.11. Statistical Analysis

All culture-based and hydrocarbon-removal experiments were performed in triplicate, and data are presented as mean ± standard deviation (SD). Normality and homogeneity of variance were evaluated using the Shapiro–Wilk test and Levene’s test, respectively. For datasets satisfying the assumptions, differences among treatments were analyzed by one-way analysis of variance (ANOVA) followed by Tukey’s multiple-comparison test. Specifically, this procedure was applied to comparisons of Shannon diversity, endpoint OD_600_ values in the saline hydrocarbon-utilization assays, hydrocarbon-removal efficiencies among individual isolates, and C_24_ removal by the three-strain consortium. Because the hydrocarbon-removal data shown in Figure S4 did not meet the assumption of normality, differences among treatments in this experiment were analyzed using the Kruskal–Wallis test followed by Dunn’s multiple-comparison test. Differences were considered statistically significant at *p* < 0.05. Statistical analyses were performed using R (version 4.3.1) and GraphPad Prism 9.

## 3. Results

### 3.1. Microbial Community Profiles of Paired Crude-Oil and Produced-Water Samples

The produced-water samples were characterized by moderate-to-high salinity and variable organic loads, with TDS ranging from 14.96 to 25.50 g/L, TOC from 76.0 to 849.1 mg/L, and TPH from 149 to 846 mg/L (Table S2). Bacterial community profiling of the three produced-water samples (W1–W3) and their paired crude-oil samples (O1–O3) subsequently revealed substantial differences in microbial diversity and taxonomic composition.

**Figure 2:**
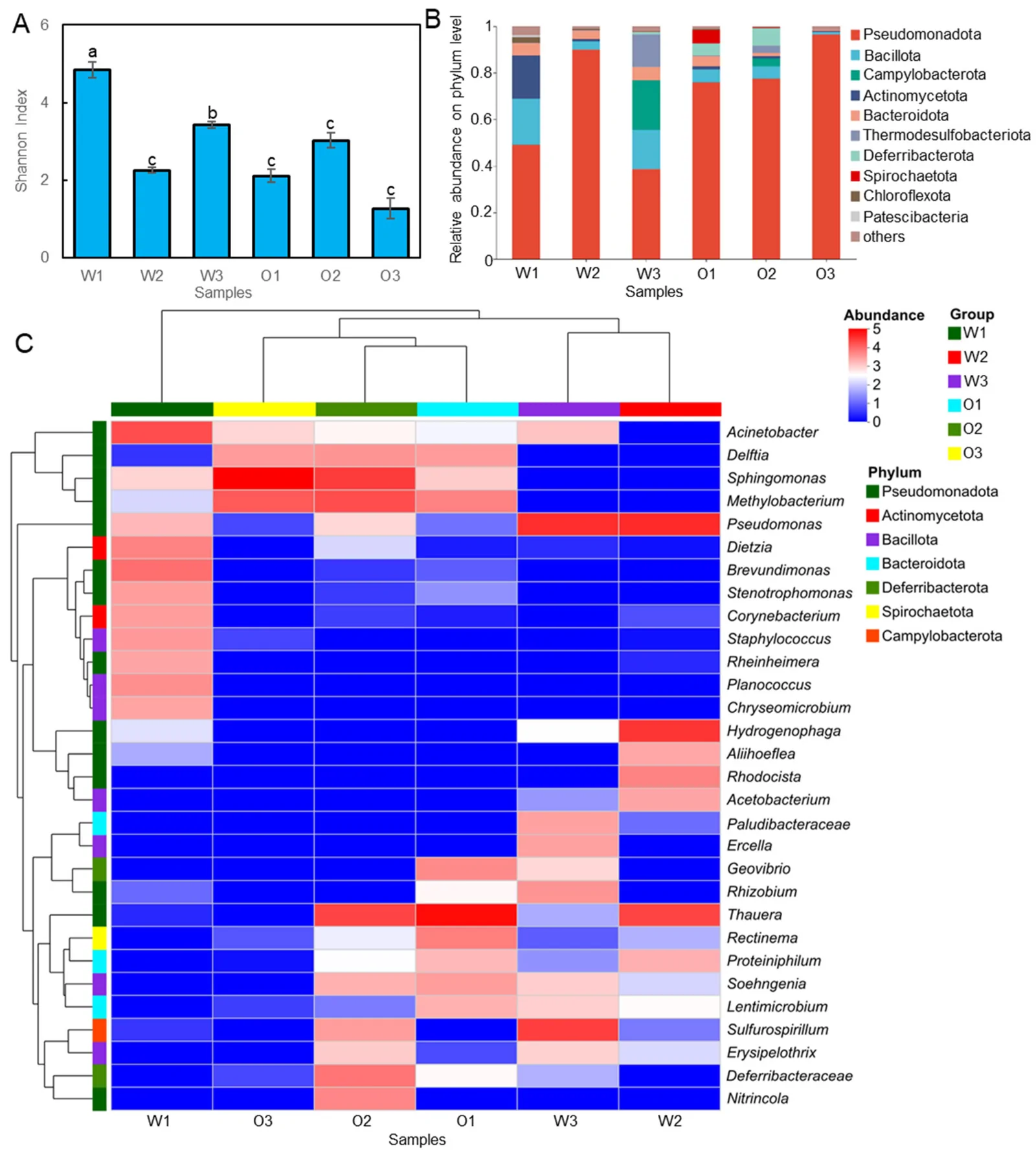
Shannon diversity index (**A**), phylum-level community composition (**B**), and genus-level hierarchical clustering heatmap (**C**) of the six samples. W1–W3 represent produced-water samples, whereas O1–O3 represent the corresponding crude-oil samples. Different lowercase letters above the bars indicate statistically significant differences among samples at *p* < 0.05.

The Shannon diversity index ranged from 1.27 to 4.85 (Figure 2A). Sample W1 exhibited the highest microbial diversity (4.85 ± 0.21), followed by W3 (3.43 ± 0.08) and O2 (3.03 ± 0.20), whereas O3 showed the lowest diversity (1.27 ± 0.27). Multiple comparisons showed that W1 had significantly higher diversity than all other samples, while W3 also exhibited significantly higher diversity than W2, O1, O2, and O3 (*p* < 0.05). No significant differences were detected among W2, O1, O2, and O3. Notably, W1 and W3 exhibited higher Shannon diversity than their corresponding crude-oil samples O1 and O3, respectively, whereas this pattern was not observed for the W2–O2 pair, indicating substantial heterogeneity among different wells and sample matrices.

At the phylum level, Pseudomonadota predominated across all six samples, although its relative abundance varied markedly among samples (Figure 2B). W2, O1, O2, and O3 were strongly dominated by Pseudomonadota, whereas W1 and W3 contained higher proportions of Actinomycetota, Bacillota, Bacteroidota, Deferribacterota, and several minor phyla. Genus-level profiles were highly heterogeneous among samples (Figure 2C). *Sphingomonas* and *Thauera* reached particularly high relative abundances in individual samples, with maximum values of approximately 86.7% and 64.2%, respectively. *Acinetobacter*, *Delftia*, *Pseudomonas*, *Dietzia*, *Methylobacterium*, and *Brevundimonas* also showed pronounced sample-specific distributions.

Several dominant or recurrent genera identified in the amplicon dataset, including *Pseudomonas*, *Sphingomonas*, *Acinetobacter*, and *Dietzia*, contain well-established hydrocarbon-degrading members, providing an ecological context for the subsequent cultivation and functional screening.

Beta-diversity analysis further indicated substantial heterogeneity among the six bacterial communities. PCoA suggested a tendency toward matrix-associated differences in community structure; however, ANOSIM did not indicate a statistically significant overall difference between produced-water and crude-oil communities (R = 0.5185, *p* = 0.10, Figure S1A). Core-microbiome analysis identified 20 core genera in crude-oil samples and 12 in produced-water samples, with only one genus shared between the two matrices (Figure S1B; Table S3). Exploratory LEfSe analysis identified several taxa showing differential representation between the two matrix types (Figure S1C).

### 3.2. Diversity and Taxonomic Composition of Cultured Isolates

**Figure 3:**
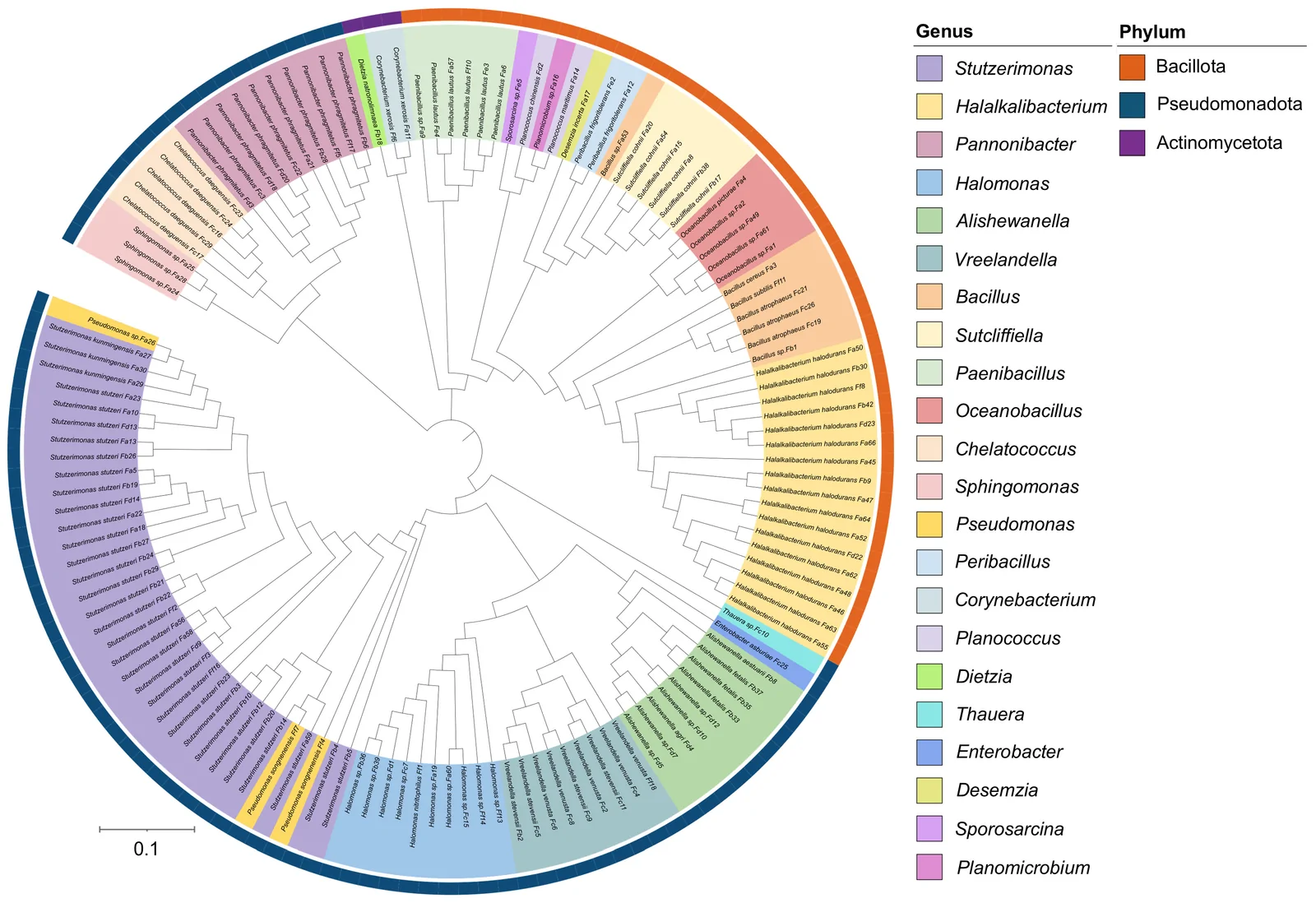
Phylogenetic relationships and genus-level composition of the cultured bacterial collection. Maximum likelihood phylogenetic tree of 135 bacterial isolates recovered from the six oilfield samples.

A total of 135 bacterial isolates were recovered from the paired crude-oil and produced-water samples and identified by 16S rRNA gene sequencing, representing three phyla, 15 families, and 22 genera (Figure 3, Table S4). Pseudomonadota and Bacillota dominated the cultured collection, whereas Actinomycetota accounted for a smaller proportion. At the genus level, *Stutzerimonas* was the most frequently recovered taxon with 33 isolates, followed by *Halalkalibacterium* with 17 isolates, and *Pannonibacter* and *Halomonas* with 10 isolates each. The taxonomic composition also varied among sample sources, indicating considerable heterogeneity in the cultivable bacterial resources associated with the different oilfield samples.

**Figure 4:**
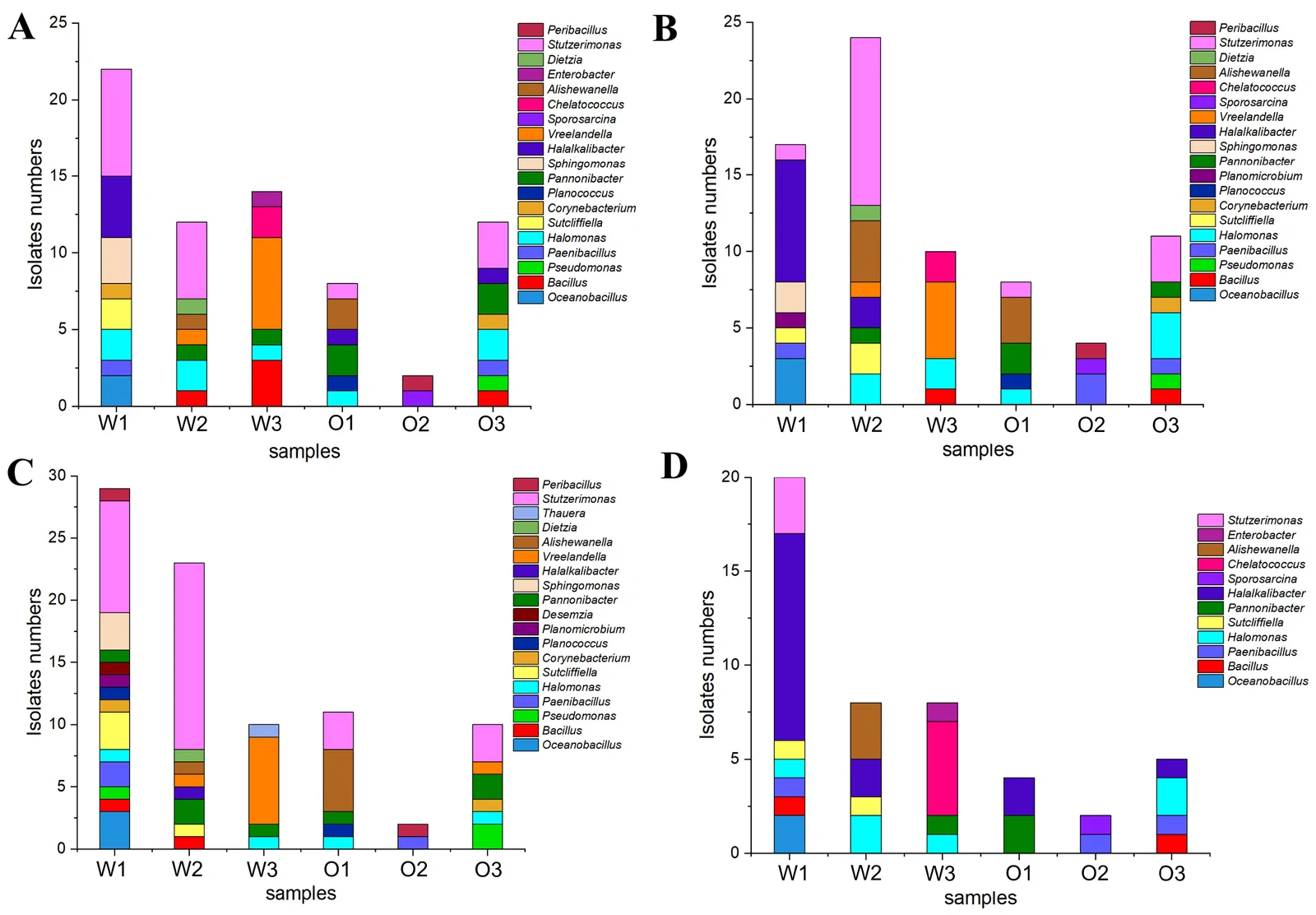
Genus-level composition of isolates showing OD_600_ > 0.3 under hydrocarbon-utilization screening with at least one tested hydrocarbon (**A**) or halotolerance screening in LB containing 6% NaCl (**B**). Genus-level composition of isolates recovered at 30 °C (**C**) and 45 °C (**D**).

Primary hydrocarbon-utilization screening identified 70 isolates that reached an OD_600_ of at least 0.3 with C_24_, naphthalene, phenanthrene, or fluoranthene as the sole carbon source. These isolates were distributed across diverse genera, including *Halomonas*, *Stutzerimonas*, *Sporosarcina*, *Sutcliffiella*, *Bacillus*, *Corynebacterium*, *Pannonibacter*, *Oceanobacillus*, *Vreelandella*, *Alishewanella*, *Paenibacillus*, and *Halalkalibacterium* (Figure 4A). Halotolerance was also widespread within the collection: 74 isolates tolerated at least 6% NaCl, and 22 retained growth at 10% NaCl, corresponding to approximately 16.3% of all cultured isolates (Figure 4B). Among the screened strains, 18 combined hydrocarbon-utilizing potential with halotolerance, while 24 combined hydrocarbon utilization with growth at elevated temperature. Oil-spreading activity was detected in a smaller subset of isolates, with 11 producing clear zones larger than 20 mm and six exceeding 30 mm (Table S4). Together, these results revealed a cultivable bacterial resource containing multiple strains with combinations of hydrocarbon utilization and environmental-stress tolerance.

Cultivation temperature strongly influenced the composition of the recovered isolates. Eighty-five isolates were obtained at 30 °C and 50 at 45 °C. *Stutzerimonas* predominated among isolates recovered at 30 °C, whereas *Halalkalibacterium*, *Halomonas*, *Bacillus*, and *Chelatococcus* were more strongly represented among those recovered at 45 °C (Figure 4C,D). Further thermotolerance testing of the 50 isolates initially recovered at 45 °C showed that 43 retained growth at 50 °C, whereas six did not grow above 45 °C. *Halomonas* sp. Fc15 was the only isolate capable of growth at 55 °C, and none of the isolates grew at 60 °C. These results indicate that cultivation at elevated temperature enabled the recovery of a substantial fraction of bacteria with moderate thermotolerance, together with a small number of more strongly temperature-tolerant isolates.

The three cultivation media also recovered different taxonomic subsets of the oilfield bacterial community. LB medium yielded 67 isolates representing 19 genera, whereas G3 medium recovered 53 isolates from 17 genera (Table S5). In contrast, the high-salinity G2 medium yielded only 15 isolates representing 6 genera, with *Halalkalibacterium*, *Oceanobacillus*, and *Halomonas* predominating. *Stutzerimonas* was the dominant genus recovered on LB and was also abundant in G3 cultures, whereas *Pannonibacter* and *Alishewanella* were more strongly represented in G3 medium. The narrower taxonomic recovery observed with G2 was consistent with the stronger selective conditions imposed by the high-salinity medium.

### 3.3. Complementarity Between Amplicon Sequencing and Culture-Dependent Isolation

**Figure 5:**
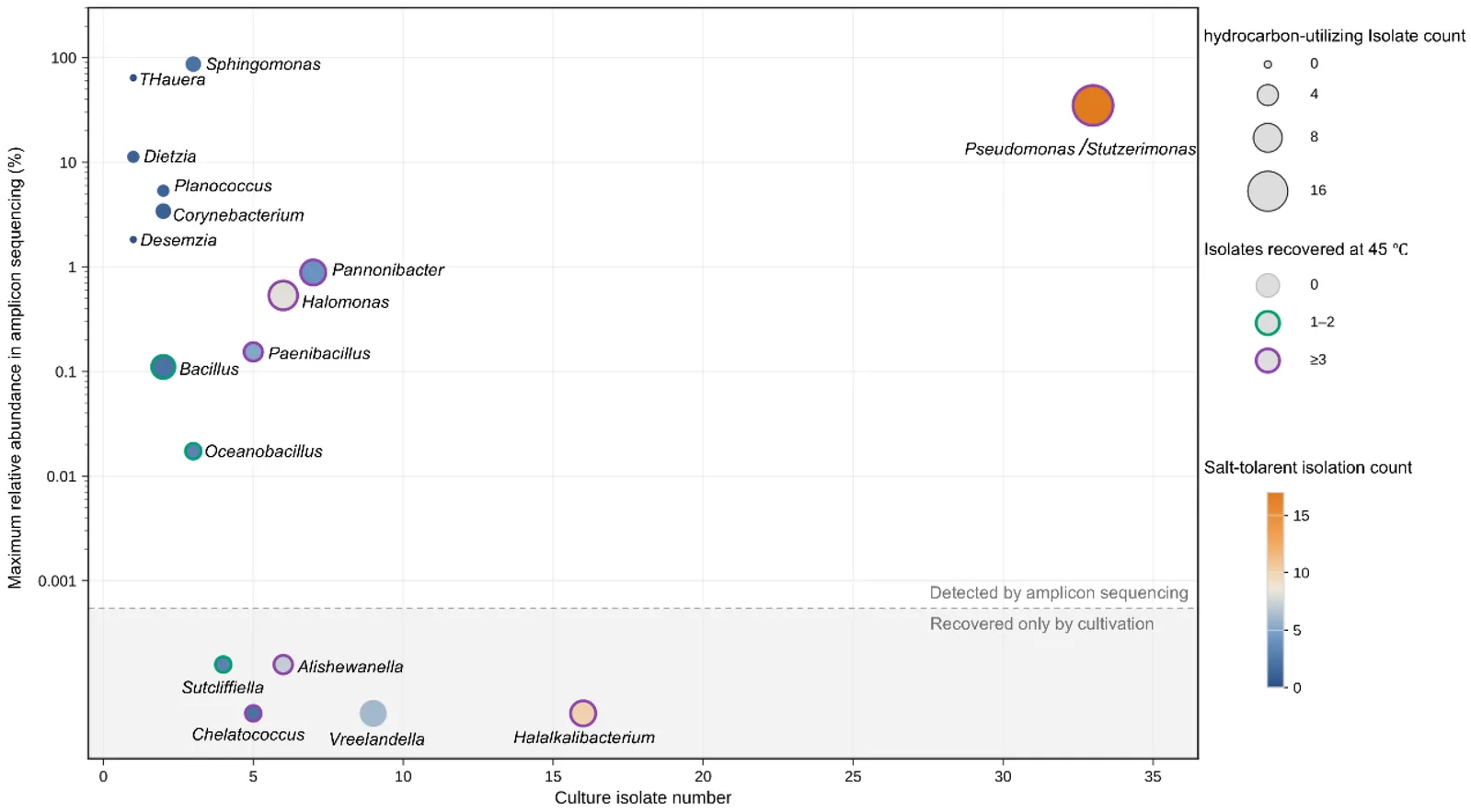
Comparison of amplicon sequencing and culture-dependent isolation at the genus level. Bubble size indicates the number of hydrocarbon-utilizing isolates, bubble color indicates the number of halotolerant isolates, and the outline indicates the number of isolates recovered at 45 °C.

To compare environmental representation with cultivation recovery and functional phenotypes, selected genera detected by amplicon sequencing and culture-dependent isolation are integrated in Figure 5. Marked discrepancies were evident between sequencing abundance and the number of isolates recovered.

*Sphingomonas* and *Thauera* reached maximum amplicon abundances of 86.7% and 64.2%, respectively, but yielded only three and one cultured isolates. In contrast, *Pannonibacter* and *Halomonas* each showed maximum sequencing abundances below 1% but yielded 10 isolates, including strains with hydrocarbon-utilizing and halotolerant or thermotolerant phenotypes. The *Pseudomonas/Stutzerimonas* lineage was also well represented in the cultured collection and contained numerous hydrocarbon-utilizing isolates. These patterns show that high environmental relative abundance did not necessarily correspond to high cultivation recovery under the conditions used here.

Five genera recovered from cultivation had no identical genus-level assignment in the amplicon dataset: *Chelatococcus*, *Alishewanella*, *Halalkalibacterium*, *Vreelandella*, and *Sutcliffiella*. *Chelatococcus* and *Alishewanella* were not detected at the corresponding genus level by amplicon sequencing. For *Halalkalibacterium*, *Vreelandella*, and *Sutcliffiella*, however, the discrepancy is at least partly attributable to taxonomic nomenclature: the amplicon dataset was annotated using an older reference taxonomy, whereas the isolates were assigned according to more recent classifications. Conversely, highly abundant sequencing taxa such as *Sphingomonas* and *Thauera* were only sparsely recovered by cultivation. Together, these results show that sequencing and cultivation captured different subsets of the oilfield bacterial community.

### 3.4. Screening and Multistress Phenotyping of Hydrocarbon-Utilizing Candidates

**Figure 6:**
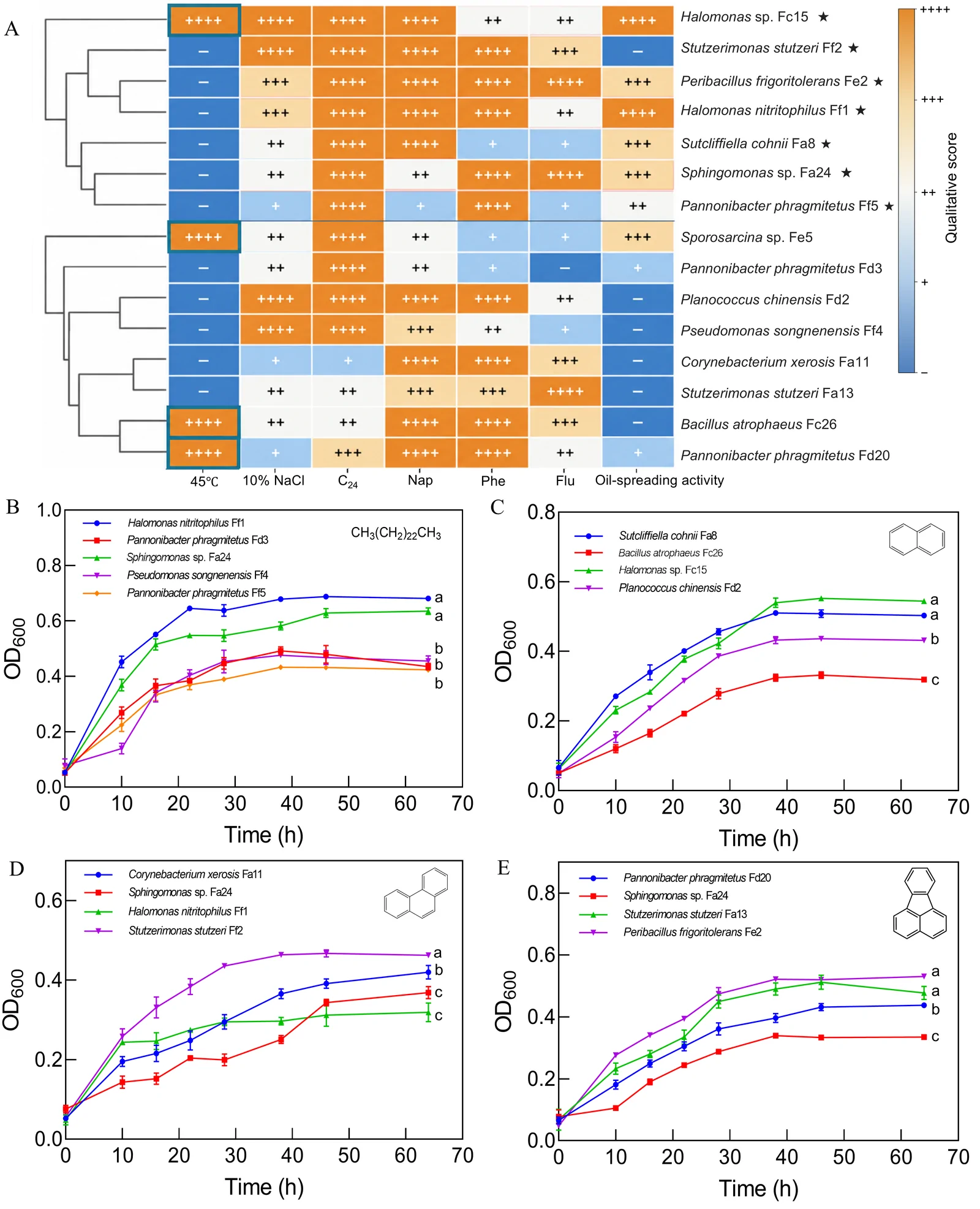
Phenotypic screening and saline growth of 15 hydrocarbon-utilizing candidates. (**A**) Integrated profiles of hydrocarbon utilization, thermotolerance, halotolerance, and oil-spreading activity. Hydrocarbon utilization was scored as −, OD_600_ < 0.1; +, 0.1–0.2; ++, 0.2–0.3; +++, 0.3–0.4; and ++++, >0.4. Thermotolerance and halotolerance indicate positive or negative growth at 45 °C and 10% NaCl, respectively. Oil-spreading activity was scored as −, <0.5 cm; +, 0.5–1.0 cm; ++, 1.0–2.0 cm; +++, 2.0–3.0 cm; and ++++, >3.0 cm. (**B**–**E**) Growth of representative isolates in MSM containing 6% NaCl with C_24_ (**B**), naphthalene (**C**), phenanthrene (**D**), or fluoranthene (**E**) as the sole carbon source at 30 °C. Asterisks indicate the seven isolates selected for subsequent analyses; blue outlines indicate isolates exhibiting both thermotolerance and halotolerance. Statistical comparisons were performed using OD_600_ values at 48 h, corresponding to the predefined screening endpoint, by one-way ANOVA followed by Tukey’s multiple-comparison test (*p* < 0.05). Different lowercase letters indicate significant differences (*p* < 0.05).

To prioritize hydrocarbon-utilizing bacteria for quantitative verification, the cultured isolates were first screened for growth in MSM containing C_24_, naphthalene, phenanthrene, or fluoranthene as the sole carbon source. Seventy isolates reached an OD_600_ of at least 0.3 with one or more hydrocarbons, and 15 isolates showing an OD_600_ above 0.4 after 48 h were selected for further characterization. These candidates differed in hydrocarbon utilization, halotolerance, thermotolerance, and oil-spreading activity (Figure 6A). *Halomonas* sp. Fc15 was the only candidate capable of growth at 55 °C, while several isolates tolerated 10% NaCl. *Halomonas nitritophilus* Ff1 showed the largest oil-spreading zone among the candidates.

To further evaluate hydrocarbon utilization under saline conditions, the 15 candidates were tested in MSM containing 6% NaCl and individual hydrocarbons. Growth remained strongly strain- and substrate-dependent. C_24_ generally supported the strongest growth, with *Halomonas nitritophilus* Ff1 and *Sphingomonas* sp. Fa24 among the best-performing isolates (Figure 6B). Distinct sets of isolates showed comparatively strong growth with naphthalene, phenanthrene, and fluoranthene (Figure 6C–E), indicating that performance under saline conditions varied considerably among both strains and substrates.

Based primarily on hydrocarbon-utilization performance, together with halotolerance, thermotolerance, oil-spreading activity, substrate-response patterns, and taxonomic representation, seven isolates were prioritized: *Sutcliffiella cohnii* Fa8, *Halomonas* sp. Fc15, *Sphingomonas* sp. Fa24, *Peribacillus frigoritolerans* Fe2, *Halomonas nitritophilus* Ff1, *Stutzerimonas stutzeri* Ff2, and *Pannonibacter phragmitetus* Ff5. These isolates retained comparatively strong hydrocarbon-utilizing capacity under 6% NaCl while representing distinct combinations of salt tolerance, temperature tolerance, and oil-spreading activity.

### 3.5. Quantitative Hydrocarbon Removal and Molecular Verification of Seven Representative Isolates

**Figure 7:**
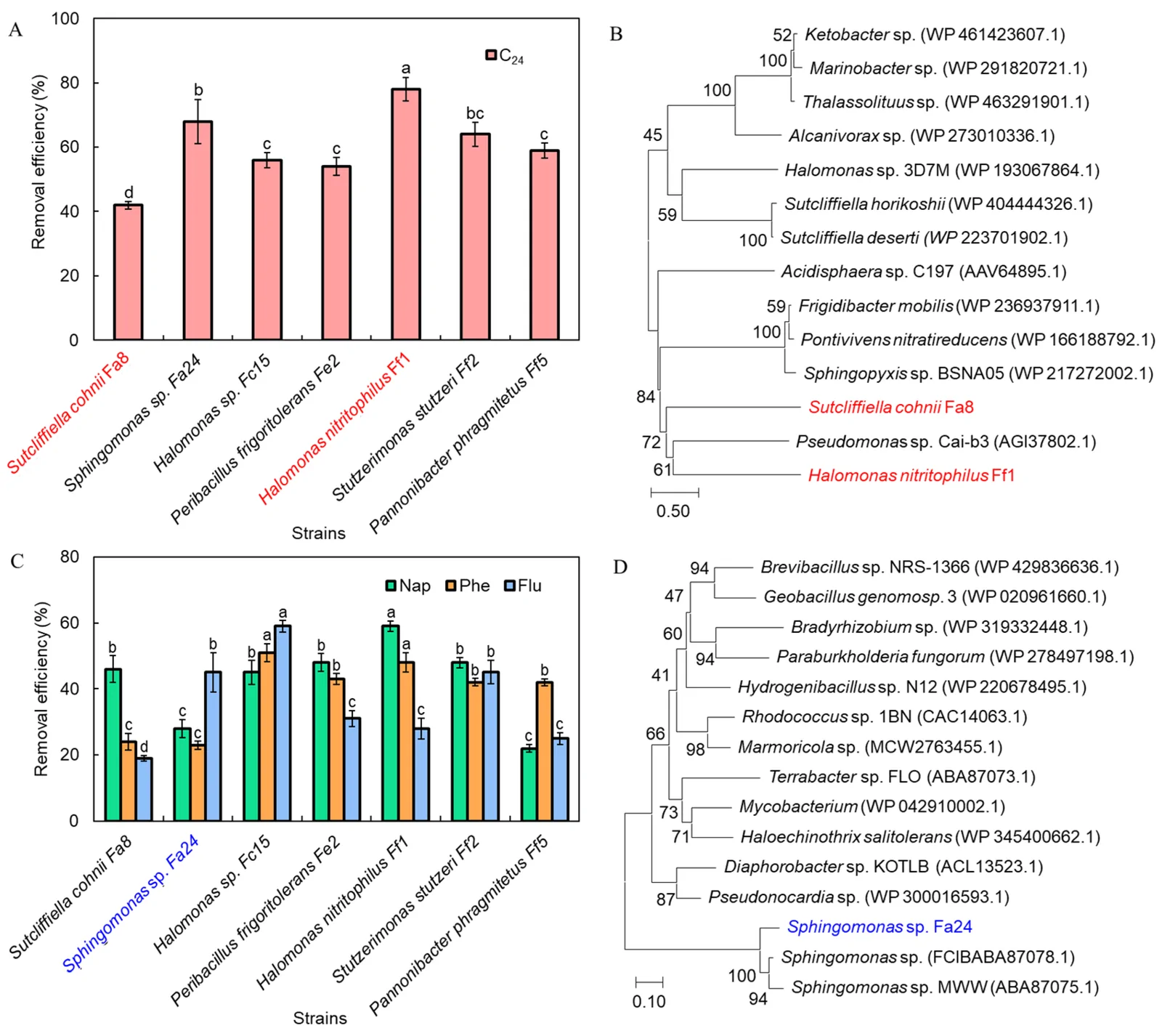
Removal efficiencies of C_24_ (**A**) and PAHs (**C**) by 7 representative isolates, and phylogenetic analyses of sequence-confirmed *alkB* (**B**) and PAH-RHDα (**D**) gene sequences from representative isolates. Removal efficiencies were calculated relative to the corresponding abiotic controls. Different lowercase letters above the bars indicate significant differences among isolates under the same hydrocarbon substrate at *p* < 0.05.

To quantitatively verify the hydrocarbon-removing capacity of the selected isolates, seven representative strains were evaluated by GC-MS under saline conditions (30 °C and 6% NaCl). All seven isolates removed the tested hydrocarbons to varying extents, but their removal profiles differed markedly among substrates (Figure 7A,C). *Halomonas nitritophilus* Ff1 exhibited the highest removal of C_24_ (78%) and naphthalene (59%), whereas *Halomonas* sp. Fc15 showed the highest removal of phenanthrene (51%) and fluoranthene (59%). *Sphingomonas* sp. Fa24 and *Stutzerimonas stutzeri* Ff2 also showed high C_24_ removal efficiencies of 68% and 64%, respectively, with Fa24 additionally removing 45% of fluoranthene. *Peribacillus frigoritolerans* Fe2 exhibited comparatively balanced removal of C_24_ (54%), naphthalene (48%), and phenanthrene (43%), whereas *Sutcliffiella cohnii* Fa8 and *Pannonibacter phragmitetus* Ff5 showed moderate removal across the tested substrates. Overall, the seven isolates displayed distinct substrate-dependent removal profiles, with some strains showing preferential activity toward C_24_ and others maintaining relatively broad activity across multiple hydrocarbons.

Five targeted functional markers associated with alkane and PAH degradation were examined by PCR followed by sequence verification, including alkB-1, ALK3, PAH-RHDα, nahAc, and C23O. Sequence validation confirmed *alkB* target identity in *Sutcliffiella cohnii* Fa8 and *Halomonas nitritophilus* Ff1, whereas PAH-RHDα was confirmed in *Sphingomonas* sp. Fa24 (Figure 7B,D). The two sequence-confirmed *alkB*-positive isolates exhibited substantial C_24_ removal, whereas Fa24, carrying a sequence-confirmed PAH-RHDα fragment, showed appreciable fluoranthene removal. However, the limited number of sequence-confirmed marker genes precluded a direct genotype–phenotype relationship across the seven isolates. Failure to obtain sequence-confirmed products for the remaining target genes may reflect primer specificity, sequence divergence, or other methodological limitations and should not be interpreted as evidence for the absence of hydrocarbon-degradation pathways.

To further assess hydrocarbon removal under combined heat and salinity stress, four thermo-halotolerant isolates, *Bacillus atrophaeus* Fc26, *Halomonas* sp. Fc15, *Pannonibacter phragmitetus* Fd20, and *Sporosarcina* sp. Fe5, were tested at 45 °C and 6% NaCl (Figure S3). All four retained hydrocarbon-removal activity, with efficiencies ranging from 14% to 57% depending on the strain and substrate. Among these isolates, only Fc15 was also included in the 30 °C quantitative assay. Its C_24_ removal remained essentially unchanged at 56–57% between 30 and 45 °C, whereas removal of the three PAHs decreased by approximately 8 to 16 percentage points at 45 °C.

Collectively, these assays identified several oilfield-derived isolates that retained substantial hydrocarbon-removal activity under saline conditions, including strains capable of simultaneous tolerance to elevated salinity and temperature. Sequence-verified catabolic markers provided additional molecular support for the observed hydrocarbon-removal phenotypes.

### 3.6. Enhanced C_24_ Removal by a Three-Strain Consortium

**Figure 8:**
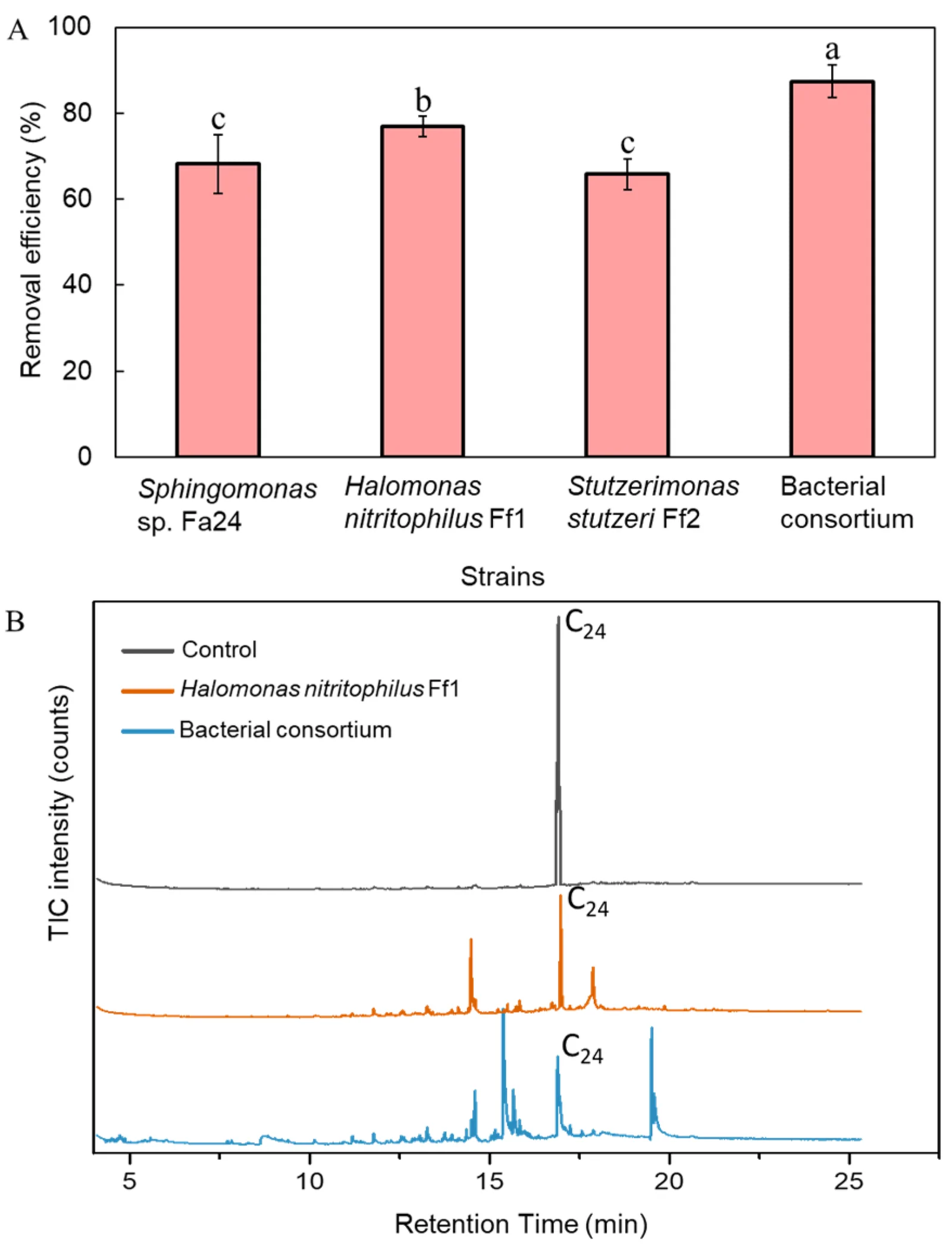
Enhanced C_24_ removal by a three-strain consortium (**A**) and representative GC-MS chromatograms (**B**). Chromatograms are shown for the uninoculated control, *Halomonas nitritophilus* Ff1, and the three-strain consortium. Removal efficiencies were calculated relative to the corresponding abiotic controls. Different lowercase letters above the bars indicate significant differences among treatments at *p* < 0.05.

The three isolates showing the highest individual C_24_ removal efficiencies, *Sphingomonas* sp. Fa24, *Halomonas nitritophilus* Ff1, and *Stutzerimonas stutzeri* Ff2, were combined in equal proportions for consortium testing. No visible pairwise antagonistic interactions were observed among Fa24, Ff1, and Ff2 under the tested conditions (Figure S5). The consortium and monocultures received the same total inoculum and were incubated at 30 °C with 6% NaCl. The consortium achieved 87.4% C_24_ removal (Figure 8A), significantly exceeding the three monocultures by 10.5–21.6 percentage points (*p* < 0.05). Representative GC-MS chromatograms showed substantially lower residual C_24_ in the consortium than in the abiotic control (Figure 8B). These results demonstrate that combining selected oilfield-derived degraders further enhanced long-chain alkane removal under saline conditions.

## 4. Discussion

Saline petroleum environments impose simultaneous constraints on microbial growth and hydrocarbon degradation, making indigenous microorganisms a useful source of adapted degraders [9,44]. In the present study, paired crude-oil and produced-water samples from the Huoshaoshan Oilfield showed marked differences in bacterial community composition, while cultivation yielded 135 isolates representing 22 genera (Figure 2A). Many isolates tolerated elevated salinity or temperature and showed hydrocarbon-utilizing or oil-spreading activity. Quantitative assays further confirmed substantial C_24_ and PAH removal under 6% NaCl, and a three-strain consortium increased C_24_ removal to 87.4% (Figure 7A,C). These results demonstrate that the Huoshaoshan Oilfield contains a diverse cultivable bacterial resource with traits relevant to hydrocarbon degradation under saline conditions.

Pseudomonadota dominated the bacterial communities in this study, while the relative abundances of genera such as *Sphingomonas*, *Acinetobacter*, *Pseudomonas*, and *Dietzia* varied markedly among samples. Two produced-water samples showed higher Shannon diversity than their paired crude oil samples, whereas the third pair showed the opposite pattern. Heterogeneous microbial distributions have also been observed in other petroleum reservoirs. In the Edvard Grieg reservoir, microbial communities differed among wells and production-associated fluids, with *Thermoanaerobacter*, *Thermococcus*, and *Halomonas* among the prevalent genera, and substantial community shifts occurring after water breakthrough [21]. The comparatively high diversity observed in some produced-water samples here may reflect the greater physicochemical heterogeneity of the aqueous phase, which contains dissolved salts, organic matter, and residual oil and can therefore provide both aqueous and oil-associated microenvironments [23,45]. Crude oil, in contrast, imposes strong hydrophobic constraints and limits the availability of dissolved nutrients. The opposite pattern observed in one sample pair indicates that these differences are not uniform across wells. Analysis of corresponding crude oil and produced water therefore provides a broader view of petroleum-associated microbial diversity than either matrix considered alone. The absence of some well-known hydrocarbon-degrading actinomycetes, such as *Rhodococcus* and *Gordonia*, may reflect site-specific community composition, low abundance, and the selective effects of the cultivation conditions used in this study.

The cultured isolates displayed substantial physiological diversity, including among closely related taxa, emphasizing the importance of strain-level functional characterization. Of the 135 isolates, 74 tolerated at least 6% NaCl and 22 grew at 10% NaCl, while *Halomonas* sp. Fc15 retained growth at 55 °C (Table S4). Hydrocarbon screening identified 15 candidates that grew well with at least one of C_24_, naphthalene, phenanthrene, or fluoranthene as the sole carbon source (Figure 6A). Subsequent GC-MS assays showed that the best isolates removed up to 78% of C_24_ and 59% of the tested PAHs under 6% NaCl without further optimization of degradation conditions (Figure 7A). For comparison, *Bacillus subtilis* HG01 degraded 77.2% of tetracosane and 55.5% of pyrene at 5% NaCl [46]. The halotolerant *Pannonibacter phragmitetus* CGMCC9175 has also been reported to degrade naphthalene, phenanthrene, and anthracene in the presence of 5% NaCl [47]. In another oilfield-derived isolate, *Halomonas* sp. C2SS100 was enriched on crude oil at 100 g/L NaCl and degraded several petroleum hydrocarbons, accompanied by increased emulsifying activity during growth on hexadecane [12]. These previous studies provide relevant benchmarks for the present isolates. More importantly, the Huoshaoshan collection contained multiple strains combining substantial hydrocarbon removal with salinity tolerance, temperature tolerance, or oil-spreading activity, providing a functionally diverse resource for saline bioremediation and consortium construction.

*Halomonas nitritophilus* Ff1 was particularly notable, achieving 78% C_24_ removal under 6% NaCl while also showing strong naphthalene removal and the largest oil-spreading zone among the screened isolates (Figure 7A; Table S4). Hydrocarbon degradation is well established within the genus *Halomonas*, including strains capable of hydrocarbon utilization under hypersaline conditions [12,48,49], whereas petroleum-hydrocarbon degradation by *Halomonas nitritophilus* has received much less attention at the species level. The present results therefore provide direct evidence for long-chain alkane and aromatic-hydrocarbon removal by an oilfield-derived *Halomonas nitritophilus* under saline conditions. *Sutcliffiella cohnii* Fa8 also retained hydrocarbon-removal activity at elevated salinity. Marzuki et al. reported that *Bacillus cohnii* DSM 6307, subsequently assigned to *Sutcliffiella cohnii*, degraded C_13_, C_18_, C_23_, and C_29_ alkanes in petroleum sludge, with removal ranging from 42.34% to 54.76% [50]. Reports of this species in saline oilfield degradation systems remain limited, and Fa8 extends its documented hydrocarbon-removing capacity to an oilfield-derived strain active under elevated salinity. Functional-gene PCR provided additional sequence-level evidence for selected degradation phenotypes. Sequence-confirmed *alkB* fragments were obtained from Fa8 and Ff1, whereas a PAH-RHDα fragment was confirmed in *Sphingomonas* sp. Fa24. PCR products from other strain-primer combinations could not be verified as the intended target genes, so the current data are insufficient to establish a general genotype–phenotype relationship. Negative or non-confirmed amplification also cannot demonstrate the absence of corresponding catabolic pathways because primer specificity and sequence divergence can affect PCR detection. In the present study, hydrocarbon removal was quantified from the decrease in parent-compound concentrations relative to the corresponding abiotic controls and should therefore not be interpreted as complete mineralization. The additional chromatographic peaks observed after incubation may represent intermediate transformation products, and further metabolite identification would be required to resolve the complete degradation pathways.

Comparison of amplicon sequencing and cultivation revealed marked differences in the representation of individual genera. *Sphingomonas* and *Thauera* reached maximum sequencing abundances of 86.7% and 64.2%, respectively, but yielded only three and one cultured isolates, whereas *Pannonibacter* and *Halomonas* each had maximum sequencing abundances below 1% but yielded ten isolates, including strains with hydrocarbon-utilizing and stress-tolerant phenotypes. Five genera were recovered only through cultivation, although the apparent absence of *Halalkalibacterium*, *Vreelandella*, and *Sutcliffiella* from the amplicon dataset may partly reflect taxonomic nomenclature differences. Similar discrepancies were recently reported in petroleum-contaminated soils, where isolates were recovered even from enrichment cultures in which the corresponding genus was detected at very low abundance or not detected by amplicon sequencing [26]. These results reinforce the practical value of cultivation for recovering functional strains that cannot be prioritized solely from environmental relative abundance.

The three isolates showing the highest C_24_ removal, *Sphingomonas* sp. Fa24, *Halomonas nitritophilus* Ff1, and *Stutzerimonas stutzeri* Ff2, were further evaluated as a consortium. All three retained strong C_24_ removal at 6% NaCl; Ff1 and Fa24 also showed oil-spreading activity, whereas Ff2 exhibited strong halotolerance (Figure S3). With the same total initial inoculum, the consortium removed 87.4% of C_24_, compared with 76.9% for the best-performing monoculture. A closely comparable study reported that a three-strain consortium containing *B. subtilis* HG01, *Pseudomonas putida*, and *P. aeruginosa* increased tetracosane and pyrene degradation by approximately 20% at 5% NaCl [46]. Other studies have also demonstrated improved petroleum degradation by combining salt-tolerant degraders with biosurfactant-producing bacteria. A *Dietzia* sp. CN-3-*Acinetobacter* sp. HC8-3S consortium, for example, degraded 95.8% of crude oil within 10 d and remained active over a broad salinity range [51]. More recently, a four-strain consortium containing halotolerant petroleum degraders and biosurfactant producers was successfully applied to high-salinity crude-oil-contaminated soil [8]. Because these studies used different substrates, salinities, and incubation conditions, their absolute removal percentages are not directly comparable with the present C_24_ assay. Nevertheless, together with the 87.4% removal achieved here, they support the feasibility of combining stress-tolerant petroleum isolates to enhance hydrocarbon degradation under saline conditions. The improvement observed in the present consortium may involve interstrain interactions [52], complementary substrate utilization, or improved hydrocarbon bioavailability, although the underlying mechanism remains to be determined. The recovered strains therefore represent promising candidates for saline petroleum bioremediation. Further evaluation in actual produced water and crude-oil matrices, together with genome-resolved analyses and reservoir-simulation experiments, will be necessary to assess their performance under more realistic petroleum-environment conditions and to optimize consortium composition.

Several aspects warrant further investigation. Oil-spreading activity indicated biosurfactant-related potential, but chemical characterization will be required to determine the properties of the surface-active compounds. Similarly, shotgun metagenomics or metatranscriptomics could extend the taxonomic information provided by 16S rRNA sequencing to community-level functional analysis. The sequence-confirmed *alkB* and PAH-RHDα fragments support potential initial steps in alkane and PAH degradation, and future genome-resolved and metabolite-based analyses would help clarify the complete pathways.

## 5. Conclusions

This study integrated microbial community profiling with culture-dependent isolation to characterize functional bacterial resources from paired crude-oil and produced-water samples of the Huoshaoshan Oilfield. A total of 135 isolates representing 22 genera were recovered, of which 70 showed hydrocarbon-utilizing potential in primary screening, while 74 tolerated at least 6% NaCl, and selected isolates also exhibited thermotolerance and oil-spreading activity. Quantitative assays confirmed substantial removal of C_24_ and PAHs by selected isolates under saline conditions, supported by sequence-verified *alkB* and PAH-RHDα fragments in several strains. A three-strain consortium achieved 87.4% C_24_ removal under 6% NaCl and significantly outperformed the corresponding monocultures. These results provide a characterized resource of stress-tolerant hydrocarbon degraders for further development in saline petroleum bioremediation.

## Figures and Tables

**Table 1:** Primers used for functional-gene amplification in this study.

Gene Name	Gene Description	Sequence (5’→3’)
*alkB* [38]	alkane monooxygenase	F: AAYACNGCNCAYGARCTNGG
		R: GCRTGRTGRTCNGARTGNCGYTG
ALK3 [39]	alkane hydroxylase	F: TCGAGCACATCCGCGGCCACCA
		R: CCGTAGTGCTCGACGTAGTT
PAH-RHDα [40]	PAH-ring hydroxylating dioxygenase α-subunit	F: GAGATGCATACCACGTKGGTTGGA
		R: AGCTGTTGTTCGGGAAGAYWGTGCMGTT
*nahAc* [41]	naphthalene dioxygenase large subunit	F: TGGCGATGAAGAACTTTTCC
		R: AACGTACGCTGAACCGAGTC
C23O [42]	catechol 2,3-dioxygenase	F: AAGAGGCATGGGGGCGCACCGGTTCGATCA
		R: AACAAA(AGT)GCGC(GC)GTCATGCGG

## Data Availability

The 16S rRNA amplicon sequencing data generated in this study are available in the NCBI Sequence Read Archive under BioProject PRJNA1499344 (https://www.ncbi.nlm.nih.gov/bioproject/PRJNA1499344; accessed on 4 September 2026). The 16S rRNA gene sequences of the cultured isolates are available in GenBank under accession numbers PZ692254-PZ692388, and the sequence-verified hydrocarbon-degradation-related gene fragments are available under accession numbers PZ815455-PZ815457. Additional data supporting the findings of this study are provided in the article and Supplementary Materials.

## Supplementary Materials

The following supporting information can be downloaded at: https://www.mdpi.com/article/10.3390/microorganisms14091981/s1, Table S1: Sampling information for the three crude-oil samples collected from the Huoshaoshan Oilfield; Table S2: Physicochemical characteristics of the three produced-water samples; Table S3: Core genera identified in crude-oil and produced-water samples, with their prevalence and mean relative abundance; Table S4: Characteristics of the 135 cultured bacterial isolates, including thermotolerance, halotolerance, hydrocarbon utilization, and oil-spreading activity; Table S5: Recovery of bacterial isolates using different cultivation media; Figure S1: Supplementary analyses of bacterial communities in crude-oil and produced-water samples. (A) Principal coordinate analysis (PCoA) based on Bray–Curtis dissimilarity; differences between matrix types were assessed by ANOSIM. (B) Venn diagram showing core genera identified in crude-oil and produced-water samples using a prevalence threshold of >0.8. (C) LEfSe cladogram showing taxa differentially represented between the two matrix types. W, produced-water samples; O, crude-oil samples; Figure S2: Standard curves of *n*-tetracosane (A), naphthalene (B), phenanthrene (C) and fluoranthene (D) for GC-MS quantification; Figure S3: Representative multistress phenotypes of characteristic isolates. (A) Halotolerance of *Stutzerimonas stutzeri* Ff2 under 1–10% NaCl. (B) Thermotolerance of *Halomonas* sp. Fc15 at 45–60 °C. (C) Time course of oil-spreading activity of *Halomonas nitritophilus* Ff1. (D) Representative oil-spreading assay of *Halomonas nitritophilus* Ff1; Figure S4: Removal efficiencies of *n*-tetracosane (C_24_), naphthalene, phenanthrene and fluoranthene by four thermo-halotolerant isolates at 45 °C and 6% NaCl. Removal efficiencies were calculated relative to the corresponding abiotic controls. Differences among treatments were evaluated using the Kruskal–Wallis test followed by Dunn’s multiple-comparison test (*p* < 0.05). Different lowercase letters above the bars indicate significant differences among isolates for the same hydrocarbon at *p* < 0.05; Figure S5: Pairwise cross-streak antagonism assay showing no visible antagonistic interactions among the three bacterial strains used for consortium construction.
