# Supplementary material for: Integrated Analysis of Paired Crude Oil and Produced Water Reveals Stress-Tolerant Hydrocarbon-Degrading Bacteria with Bioremediation Potential
Source: Microorganisms. 2026 Sep 8;14(9):1981. doi: 10.3390/microorganisms14091981 (PMC13609307; doi:10.3390/microorganisms14091981)
Supplement: Supplementary file 1 [file microorganisms-14-01981-s001.zip › microorganisms-4538717-supplementary.pdf]

Table legend

Table S1 Sampling information for the three crude-oil samples collected from the Huoshaoshan Oilfield.

Table S2 Physicochemical characteristics of the three produced-water samples.

Table S3 Core genera identified in crude-oil and produced-water samples, with their prevalence and mean relative abundance.

Table S4 Characteristics of the 135 cultured bacterial isolates, including thermotolerance, halotolerance, hydrocarbon utilization, and oil-spreading activity. (Provided as a separate Excel file.)

Table S5 Recovery of bacterial isolates using different cultivation media.

Table S1 Sampling information for the three crude-oil samples collected from the Huoshaoshan Oilfield.

**Sample No**	**Block**	**Well**	**Formation**	**Water cut**	**Average Buried Depth（m）**	**Formation Temperature (℃)**
O1	Huoshaoshan Production Zone 2	H1469	H1	86%	1480-1520	61-63
O2	Huoshaoshan Production Zone 1	H1380	H2	76%	1510-1550	63-65
O3	Huoshaoshan Production Zone 2	HTD2742	H4	37%	1600-1650	65-67

Table S2 Physicochemical characteristics of the three produced-water samples.

Sample **No**	TDS (g/L)	CODMn (mg/L)	TOC (mg/L)	TN (mg/L)	TP (mg/L)	TH (mg/L)	TPH (mg/L)
W1	23.445	182.0	736.40	8.072	0.374	1897.8	720
W2	25.500	116.0	849.10	1.664	0.457	2718.4	846
W3	14.960	43.2	76.0	29.608	0.006	122.1	149

Notes: TDS=total dissolved solids, CODMn=permanganate index, TOC=total organic carbon, TN=total nitrogen, TP=total phosphorus, TH=total hardness, TPH=total petroleum hydrocarbons.

Table S3 Core genera identified in crude-oil and produced-water samples, with their prevalence and mean relative abundance.

**Genus**	**Core status**	**Oil Prevalence**	**Mean relative abundance in crude oil (%)**	**Produced-water prevalence**	**Mean relative abundance in produced water (%)**
Acinetobacter	Crude-oil core	1.00	0.6783	0.67	6.9143
Agrobacterium	Crude-oil core	1.00	0.0917	0.00	0
Aquabacterium	Crude-oil core	1.00	0.728	0.33	0.0157
Blastococcus	Crude-oil core	1.00	0.0538	0.33	0.0468
Burkholderia-Caballeronia-Paraburkholderia	Crude-oil core	1.00	0.0712	0.00	0
Dechloromonas	Crude-oil core	1.00	0.1458	0.00	0
Delftia	Crude-oil core	1.00	3.7893	0.33	0.0076
Halomonas	Crude-oil core	1.00	0.1389	0.67	0.0165
Lentimicrobium	Crude-oil core	1.00	0.7808	0.67	0.5796
Methylobacterium	Crude-oil core	1.00	12.2536	0.33	0.0701
Methylorubrum	Crude-oil core	1.00	0.3262	0.33	0.0081
Pannonibacter	Crude-oil core	1.00	0.3916	0.67	0.0709
Proteiniphilum	Crude-oil core	1.00	0.7865	0.67	0.8196
Pseudomonas	Crude-oil core	1.00	0.3759	1.00	24.1494
Pseudothermotoga	Crude-oil core	1.00	0.1161	0.67	0.1537
Rectinema	Crude-oil core	1.00	2.2225	0.67	0.0384

Roseateles	Crude-oil core	1.00	0.0484	0.33	0.0563
SCADC1-2-3	Crude-oil core	1.00	0.1161	0.33	0.0119
Sphingobium	Crude-oil core	1.00	0.4072	0.33	0.5255
Sphingomonas	Crude-oil core	1.00	36.3815	0.33	0.4308
Anaerovorax	Produced-water core	0.67	0.0953	1.00	0.3802
Desulfobulbus	Produced-water core	0.33	0.0336	1.00	0.1946
Desulfomicrobium	Produced-water core	0.33	0.2985	1.00	0.0674
Dietzia	Produced-water core	0.67	0.0802	1.00	2.3411
Fervidobacterium	Produced-water core	0.67	0.0103	1.00	0.1937
Hydrogenophaga	Produced-water core	0.00	0	1.00	9.7394
Pseudothauera	Produced-water core	0.33	0.0595	1.00	0.8237
Sulfurospirillum	Produced-water core	0.33	1.124	1.00	7.8981
Tepidiphilus	Produced-water core	0.67	0.2299	1.00	0.3986
Thauera	Produced-water core	0.67	27.9495	1.00	7.2189
Thermoanaerobacter	Produced-water core	0.00	0	1.00	0.1875

Table S5 Recovery of bacterial isolates using different cultivation media.

**Isolation medium**	**No. of isolates**	**No. of genera**	**Dominant genera (relative abundance, %)**
G2	15	6	*Halalkalibacterium* (33.3); *Oceanobacillus* (20.0); *Halomonas* (20.0)
LB	67	19	*Stutzerimonas* (32.8); *Paenibacillus* (9.0); *Sutcliffiella* (7.5); *Halalkalibacterium* (7.5)
G3	53	17	*Stutzerimonas* (22.6); *Halalkalibacterium* (13.2); *Pannonibacter* (13.2); *Alishewanella* (9.4)

Figure legend

Figure S1. Supplementary analyses of bacterial communities in crude-oil and produced-water samples. (A) Principal coordinate analysis (PCoA) based on Bray–Curtis dissimilarity; differences between matrix types were assessed by ANOSIM. (B) Venn diagram showing core genera identified in crude-oil and produced-water samples using a prevalence threshold of >0.8. (C) LEfSe cladogram showing taxa differentially represented between the two matrix types. W, produced-water samples; O, crude-oil samples.

Figure S2. Standard curves of *n*-tetracosane (A), naphthalene (B), phenanthrene (C) and fluoranthene (D) for GC-MS quantification.

Figure S3. Representative multistress phenotypes of characteristic isolates. (A) Halotolerance of *Stutzerimonas stutzeri* Ff2 under 1-10% NaCl. (B) Thermotolerance of *Halomonas* sp. Fc15 at 45-60 °C. (C) Time course of oil-spreading activity of *Halomonas nitritophilus* Ff1. (D) Representative oil-spreading assay of *Halomonas nitritophilus* Ff1.

Figure S4. Removal efficiencies of *n*-tetracosane (C_{24}), naphthalene, phenanthrene and fluoranthene by four thermo-halotolerant isolates at 45 °C and 6% NaCl. Removal efficiencies were calculated relative to the corresponding abiotic controls. Differences among treatments were evaluated using the Kruskal–Wallis test followed by Dunn's multiple-comparison test ($P < 0.05$). Different lowercase letters above the bars indicate significant differences among isolates for the same hydrocarbon at $P < 0.05$.

Figure S5. Pairwise cross-streak antagonism assay showing no visible antagonistic interactions among the three bacterial strains used for consortium construction.

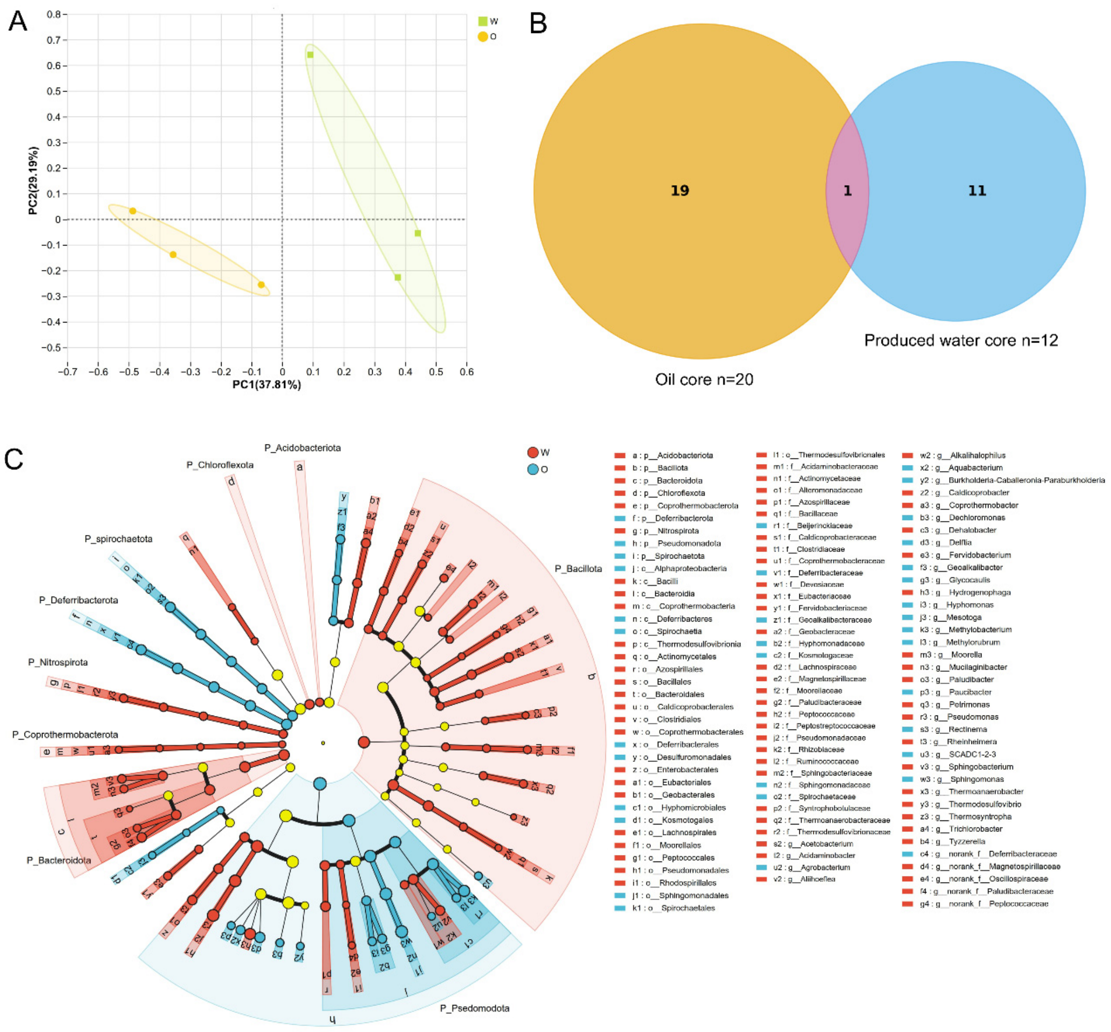

Figure S1. Supplementary analyses of bacterial communities in crude-oil and produced-water samples. (A) Principal coordinate analysis (PCoA) based on Bray–Curtis dissimilarity; differences between matrix types were assessed by ANOSIM. (B) Venn diagram showing core genera identified in crude-oil and produced-water samples using a prevalence threshold of >0.8. (C) LEfSe cladogram showing taxa differentially represented between the two matrix types. W, produced-water samples; O, crude-oil samples.

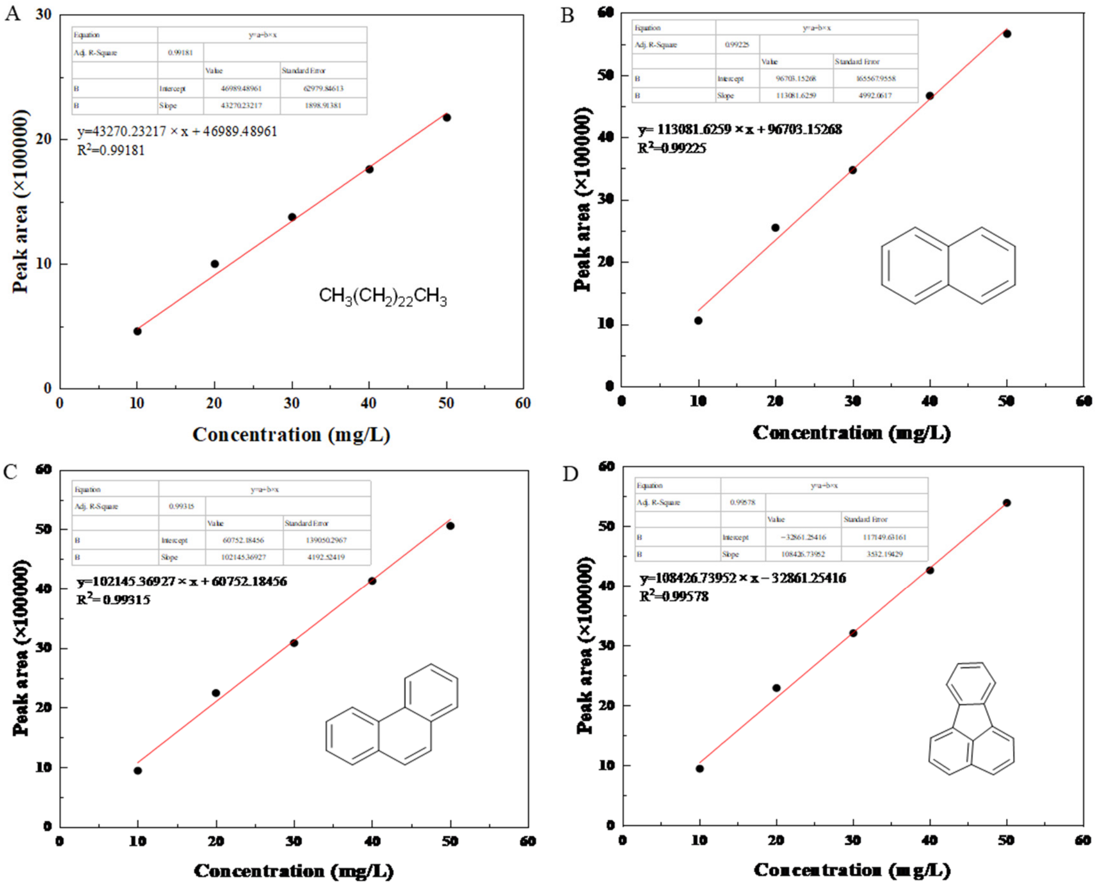

Figure S2. Standard curves of *n*-tetracosane (A), naphthalene (B), phenanthrene (C) and fluoranthene (D) for GC-MS quantification.

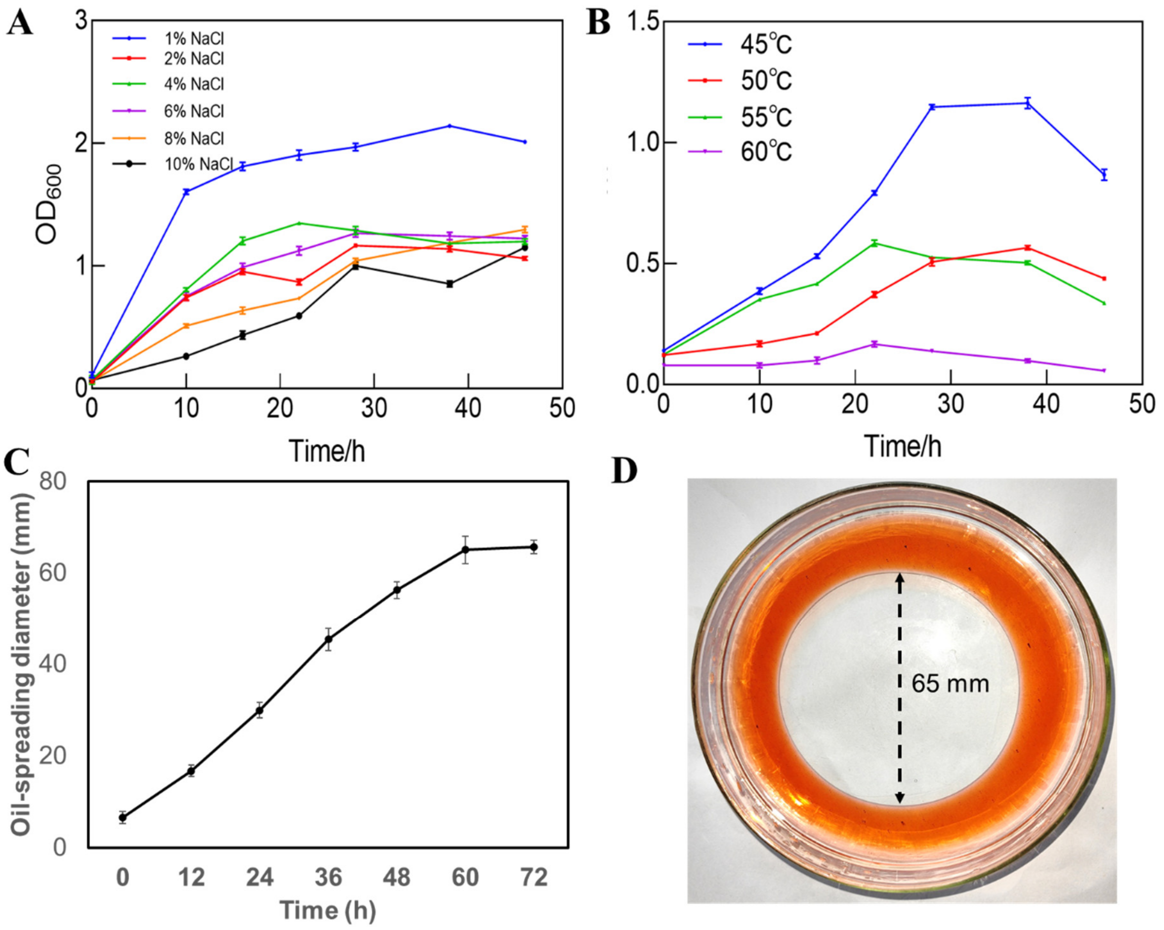

Figure S3. Representative multistress phenotypes of characteristic isolates. (A) Halotolerance of *Stutzerimonas stutzeri* Ff2 under 1-10% NaCl. (B) Thermotolerance of *Halomonas* sp. Fc15 at 45-60 °C. (C) Time course of oil-spreading activity of *Halomonas nitritophilus* Ff1. (D) Representative oil-spreading assay of *Halomonas nitritophilus* Ff1.

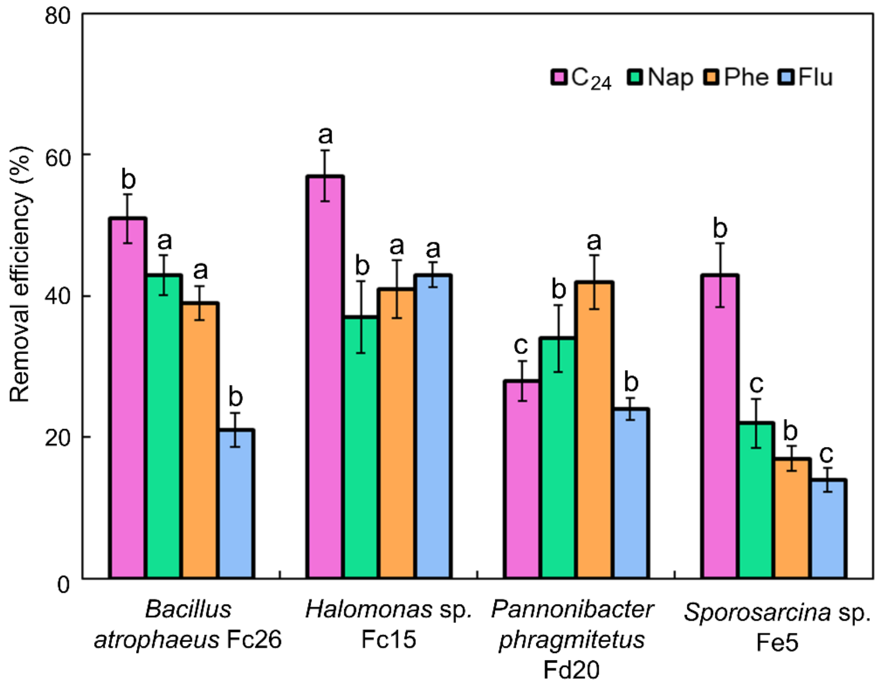

Figure S4. Removal efficiencies of *n*-tetracosane (C_{24}), naphthalene, phenanthrene and fluoranthene by four thermo-halotolerant isolates at 45 °C and 6% NaCl. Removal efficiencies were calculated relative to the corresponding abiotic controls. Differences among treatments were evaluated using the Kruskal–Wallis test followed by Dunn's multiple-comparison test ($P < 0.05$). Different lowercase letters above the bars indicate significant differences among isolates for the same hydrocarbon at $P < 0.05$.

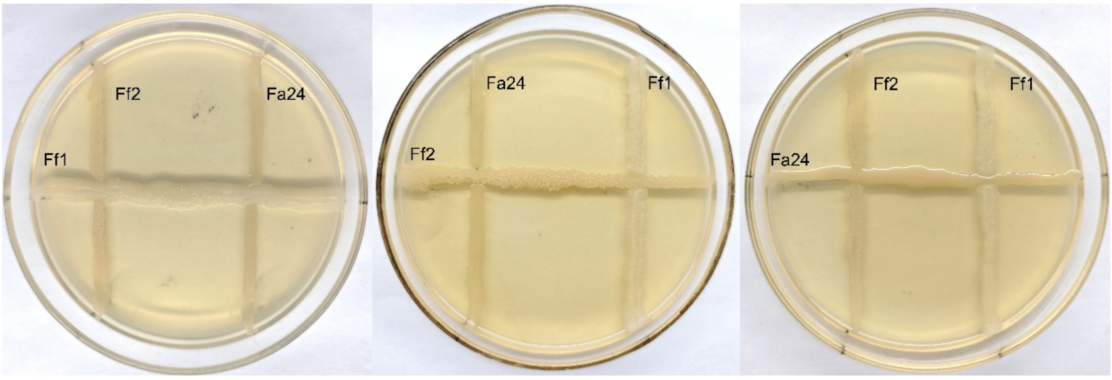

Figure S5. Pairwise cross-streak antagonism assay showing no visible antagonistic interactions among the three bacterial strains used for consortium construction.
